# Identification of Distinct Clinical Phenotypes of Heterogeneous Mechanically Ventilated ICU Patients Using Cluster Analysis

**DOI:** 10.3390/jcm12041499

**Published:** 2023-02-14

**Authors:** Xuanhui Chen, Jiaxin Li, Guangjian Liu, Xiujuan Chen, Shuai Huang, Huixian Li, Siyi Liu, Dantong Li, Huan Yang, Haiqing Zheng, Lianting Hu, Lingcong Kong, Huazhang Liu, Abdelouahab Bellou, Liming Lei, Huiying Liang

**Affiliations:** 1 Medical Big Data Center, Guangdong Provincial People’s Hospital (Guangdong Academy of Medical Sciences), Southern Medical University, Guangzhou 510080, China; 2 Department of Intensive Care Unit of Cardiac Surgery, Guangdong Cardiovascular Institute, Guangdong Provincial People’s Hospital (Guangdong Academy of Medical Sciences), Guangzhou 510080, China; 3 Shenzhen Dymind Biotechnology Co., Ltd., Shenzhen 518000, China; 4 Institute of Sciences in Emergency Medicine, Guangdong Provincial People’s Hospital (Guangdong Academy of Medical Sciences), Southern Medical University, Guangzhou 510080, China; 5 Department of Emergency Medicine, Wayne State University School of Medicine, Detroit, MI 48201, USA

**Keywords:** mechanical ventilation, cluster analysis, clinical phenotypes, critical care

## Abstract

This retrospective study aimed to derive the clinical phenotypes of ventilated ICU patients to predict the outcomes on the first day of ventilation. Clinical phenotypes were derived from the eICU Collaborative Research Database (eICU) cohort via cluster analysis and were validated in the Medical Information Mart for Intensive Care (MIMIC-IV) cohort. Four clinical phenotypes were identified and compared in the eICU cohort (n = 15,256). Phenotype A (n = 3112) was associated with respiratory disease, had the lowest 28-day mortality (16%), and had a high extubation success rate (~80%). Phenotype B (n = 3335) was correlated with cardiovascular disease, had the second-highest 28-day mortality (28%), and had the lowest extubation success rate (69%). Phenotype C (n = 3868) was correlated with renal dysfunction, had the highest 28-day mortality (28%), and had the second-lowest extubation success rate (74%). Phenotype D (n = 4941) was associated with neurological and traumatic diseases, had the second-lowest 28-day mortality (22%), and had the highest extubation success rate (>80%). These findings were validated in the validation cohort (n = 10,813). Additionally, these phenotypes responded differently to ventilation strategies in terms of duration of treatment, but had no difference in mortality. The four clinical phenotypes unveiled the heterogeneity of ICU patients and helped to predict the 28-day mortality and the extubation success rate.

## 1. Introduction

A mechanical ventilator is one of the most commonly used pieces of medical equipment in intensive care units (ICUs), delivering adequate oxygen to patients’ lungs and removing carbon dioxide. Approximately 40% of ICU patients require mechanical ventilation at any given hour [1], and thus mechanical ventilators can be in short supply when the number of critically ill patients increases, such as during the COVID-19 pandemic [2]. Although a ventilator can save lives, prolonged use of mechanical ventilation has risks including severe comorbidities, ventilator dependency, wastage of ICU resources, and additional costs [3]. Additionally, extubation failure is significantly associated with higher mortality, a longer length of stay in the ICU, and a higher likelihood of developing ventilator-associated complications [4]. Therefore, determining the optimal time to wean patients off mechanical ventilation has been a major challenge for intensivists. One of the most common predictors of successful weaning off mechanical ventilation is the rapid shallow breathing index (RSBI) during spontaneous breathing trials (SBTs) [5,6]. Many studies have tried to find better predictors or machine learning models to predict extubation failure that could be applied to every ventilated patient [7,8]. However, general prediction models have failed to achieve high accuracy for clinical practice due to the heterogeneous and dynamically changing nature of diseases in critical care. Therefore, the identification of homogeneous phenotypes is the first step towards general and accurate prediction.

Different phenotypes might respond differently to treatment strategies. Positive end-expiratory pressure (PEEP) is the pressure to prevent alveolar collapse [9]. It has been reported that the use of higher PEEP might be beneficial to patients with heart failure and pulmonary edema, but it is unlikely to improve clinical outcomes in unselected patients with acute respiratory distress syndrome [10]. Therefore, it is clinically relevant to investigate the therapeutic response of patients with different phenotypes to higher or lower PEEP strategies.

In recent years, artificial intelligence (AI) and machine learning (ML) have been widely applied in health-related research, including in the prediction of clinical outcomes in critically ill mechanically ventilated patients [11,12]. Clustering analysis is an unsupervised machine learning task that automatically discovers natural groupings in data, which could be helpful in identifying the underlying patterns in patients compared to simple stratification. Four clinical phenotypes for sepsis were derived which correlated with host–response patterns and clinical outcomes by using K-means clustering analysis [13]. Four subpopulations with distinct patterns of organ dysfunction in septic patients were identified by using hierarchical clustering [14]. Similarly, three clinical phenotypes of acute respiratory distress syndrome (ARDS) have been identified with distinct clinical characteristics and outcomes [15]. However, these studies only focused on identifying phenotypes in one type of heterogeneous condition. In another clinical study of mechanical ventilation in critically ill patients, five clinical phenotypes were correlated with different disease severities and clinical outcomes were derived [16]. However, the authors only analyzed two single-center datasets and the variables relevant to mechanical ventilation.

In this work, we aim to derive the different clinical phenotypes of heterogeneous mechanically ventilated ICU patients to help intensivists to predict the outcome of extubation on the first day of mechanical ventilation for each patient. We hypothesized that phenotypes could be derived from routinely collected clinical data by using clustering analysis and that these phenotypes would be associated with different clinical characteristics and outcomes. We hypothesized that the identification of these clinical phenotypes could help physicians provide early warning and implement personalized treatment strategies, resulting in lower mortality and better prognosis. 

## 2. Materials and Methods

### 2.1. Study Design

The study was a retrospective analysis based on two large freely available ICU databases, the eICU Collaborative Research Database (eICU) [17] and the Medical Information Mart for Intensive Care (MIMIC-IV) [18], both accessible through PhysioNet [19]. In the first step, we trained and developed a clustering model to identify clinical phenotypes by using the eICU dataset as a derivation cohort. Second, we tested and validated the clustering analysis results by using the MIMIC-IV dataset as the validation cohort. Finally, we summarized and compared the characteristics and clinical outcomes across different clinical phenotypes.

### 2.2. Population

One of the databases we used was the eICU database, which is a multicenter ICU database with over 200,000 admissions for over 130,000 different patients admitted to one of 335 ICUs at 208 hospitals in the United States between 2014 and 2015 [17]. The other database we used was the MIMIC-IV database, which includes over 380,000 patients admitted to the ICU at the Beth Israel Deaconess Medical Center between 2008 and 2019 [18]. Both databases include patient demographics, vital signs, laboratory measurements, diagnoses, medications and treatments, and provider observations and notes. 

We identified all adult ICU patients (aged ≥ 18 years old) who underwent invasive mechanical ventilation for more than 24 h, as suggested by [20], thus excluding the majority of routine postoperative ventilation events. We also filtered out patients with chronic ventilator dependency (ventilated for more than 60 days). For patients with multiple ventilation events per ICU stay, only the first ventilation event was retained. Patients with more than 30% of missing variables were excluded. A patient’s weaning event from the ventilator was considered successful if there was no reintubation or death within 48 h after weaning; otherwise, it was considered a failure event. We applied the same filtering criteria to both databases: for eICU, we filtered out 15,256 ventilation events with a failure rate of 23.1%; for MIMIC-IV, we identified 10,813 ventilation events, with a weaning failure rate of about 26.3%. The baseline characteristics of both cohorts are shown in Table A1. The multicenter eICU cohort was used as the cluster derivation cohort and the single-center MIMIC-IV cohort was used as the cluster validation cohort. 

### 2.3. Selection of Clinical Variables

We selected 40 candidate variables, including patient demographics, vital signs, blood gas analysis, laboratory measurements, scores, and mechanical ventilator parameters. Clinical variables were selected from each dataset based on their availability, and variables with more than 30% of missing values were removed. Variables with correlations greater than 0.7 were eliminated. The 24 clinical variables selected for phenotyping were age, body mass index (BMI), heart rate, respiratory rate, systolic blood pressure, oxygen saturation (SpO2), temperature, blood urea nitrogen (BUN), creatinine, platelet count, red blood cell (RBC) count, white blood cell (WBC) count, glucose, potassium, sodium, calcium, pH, bicarbonate, partial pressure of oxygen (PaO2), partial pressure of carbon dioxide (PaCO2), Glasgow Coma Scale (GCS) score, tidal volume, positive end-expiratory pressure (PEEP), and fraction of inspired oxygen (FiO2). For each variable, we removed outlier values based on a predefined outlier range and calculated the mean value during the first 24 h of mechanical ventilation. 

### 2.4. Statistical Analysis

To prepare the data for phenotyping, we first assessed the distribution, correlation, and missing values of the selected clinical variables. Data cleaning was performed and missing values were imputed by using multiple imputation with chained equations (MICE), which would introduce less bias than the mean imputation. After examining the distribution of the variables, logarithmic transformation was applied to non-normal distributions, followed by standardization. Finally, principal component analysis (PCA) was applied to reduce the dimensionality of the dataset, and the number of principal components which explained at least 80% of the variance in the data was selected. In this study, 14 PCA components were selected as inputs to the clustering model. 

K-means clustering is one of the most popular clustering models for grouping similar data points and discovering underlying patterns. Starting with a target number *k*, *k* randomly selected data points are assigned as cluster centroids; each data point is then allocated to the nearest cluster, and then new cluster centroids are calculated by averaging the data within each cluster. The clustering process is repeated until the centroids are stabilized. In this study, K-means clustering was used to assign patients to different clusters. Several clustering performance evaluation methods such as elbow plot, silhouette coefficient, cluster sizes, and meaningfulness were used to determine the optimal number of clusters *k*. The 4-cluster (*k* = 4) model was adopted in this study and the clusters were analyzed and summarized by their characteristics to form different phenotypes. 

To compare and visualize the optimal phenotype results, a baseline table was used to compare the characteristics between each phenotype with medians, interquartile ranges (IQRs), means, and standard deviations (SDs), and counts and percentages were presented appropriately. The chi-square test was used for categorical variables and the Kruskal–Wallis test was used for continuous variables with non-normal distributions. Phenotypes were visualized in two dimensions using PCA components, and chord diagrams were used to show the relationship between each phenotype and abnormal clinical variables. Cumulative mortality in the first 28 days was also compared between phenotypes. Analyses were performed using Python 3.8.5 (Python Software Foundation).

## 3. Results

### 3.1. Patients in the Study

A total of 15,256 mechanically ventilated patients from the eICU and 10,813 from the MIMIC-IV database were included in this study. The eICU patients were included in the model derivation cohort and the MIMIC-IV patients were included in the model validation cohort. The selected clinical variables were compared between the two cohorts using the Kruskal–Wallis test, and the results are shown in Appendix A Table A2.

### 3.2. Derivation of Clinical Phenotypes

**Figure 1:**
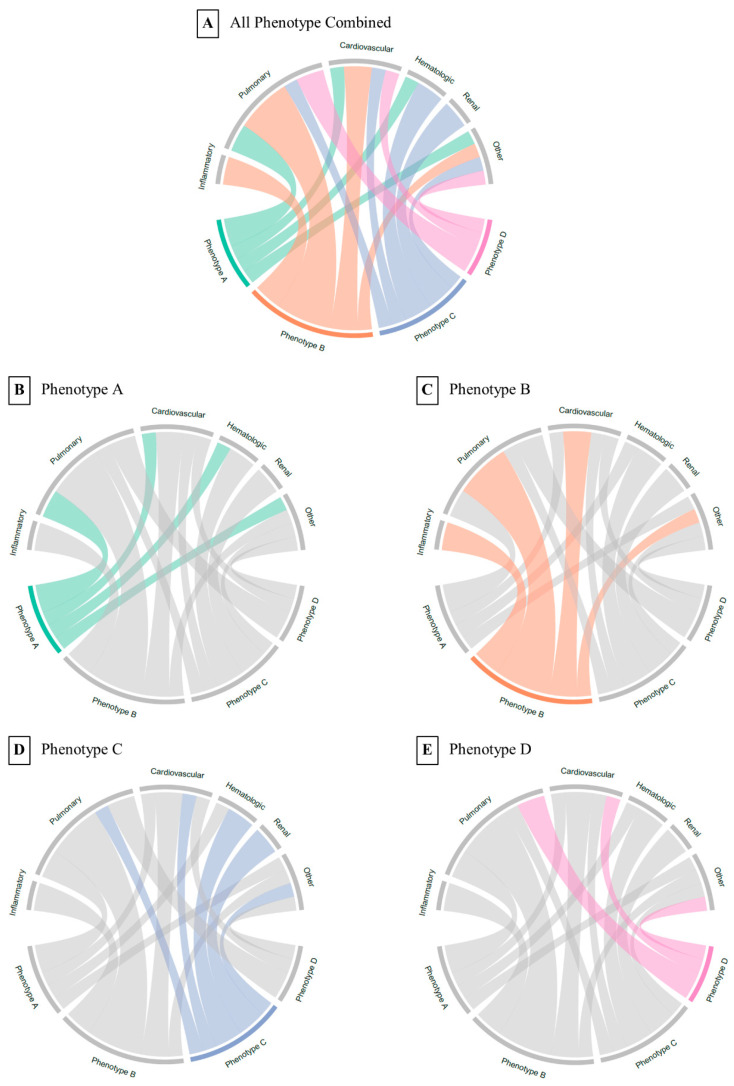
Chord diagrams showing abnormal clinical variables by phenotype. In (**A**), the ribbons connect from one phenotype to one variable group if the phenotype mean is greater or less than the overall mean for the entire cohort. For example, phenotype (**C**) is more likely to have patients with abnormal renal function. In (**B**–**E**), each phenotype is highlighted separately for ease of interpretation.

**Figure A1:**
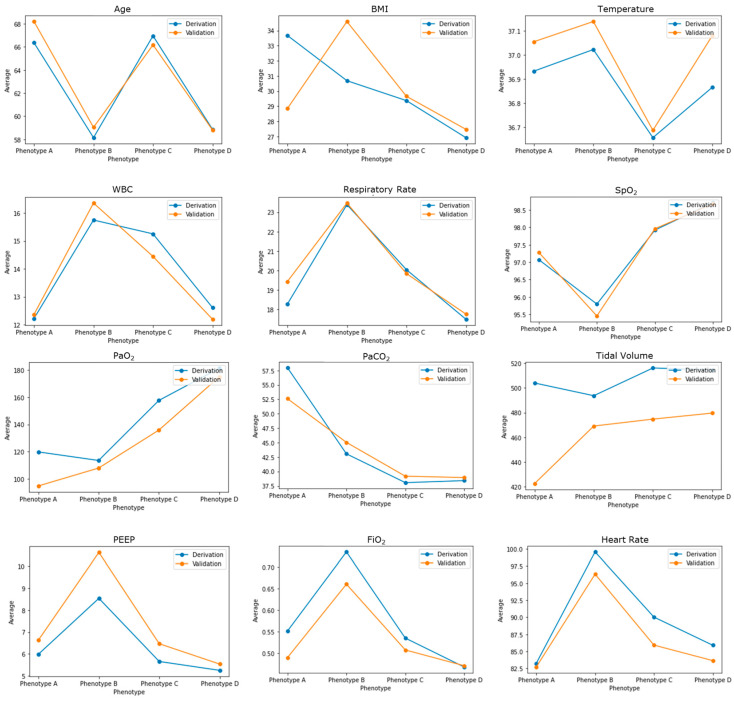

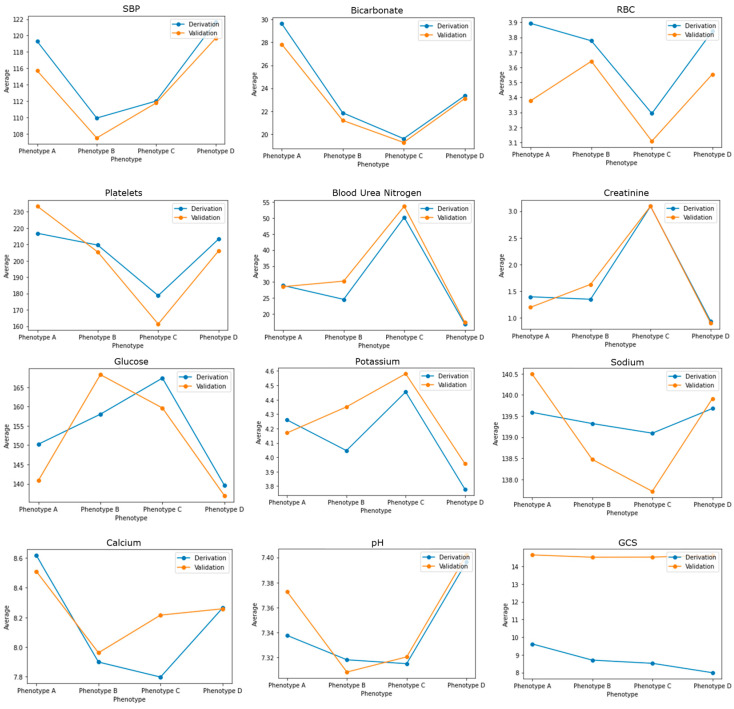
Descriptive plots of phenotype variables. Abbreviations: WBC, white blood cell; SpO_2_, oxygen saturation; PaO_2_, partial pressure of oxygen; PaCO_2_, partial pressure of carbon dioxide; PEEP, positive end expiratory pressure; FiO_2_, the fraction of inspiration oxygen; SBP, systolic blood pressure; RBC, red blood cell; GCS, Glasgow Coma Scale Score.

According to the clustering performance evaluation methods, the optimal number of clusters was determined to be four. The derived phenotypes were visualized by two- and three-dimensional plots using PCA components as coordinates. The clinical characteristics and sizes of the four derived phenotypes of A, B, C, and D are presented in Table 1 and Figure 1. All of the continuous clinical variables were not normally distributed, so the Kruskal–Wallis test was used to compare between phenotypes, and the *p*-values were all significant (<0.001). Phenotype A had the smallest population and phenotype D had the largest population. Phenotype A patients were older, had a lower PaO_2_ and a higher PaCO_2_, and were more likely to have respiratory disease; Phenotype B patients had faster heart rate and respiratory rates, higher temperature and white blood cell counts, and were more likely to have cardiovascular disease; Phenotype C patients had lower bicarbonate, pH, and red blood cell counts and higher blood urea nitrogen and creatinine, suggesting that they were more likely to have renal dysfunction; Phenotype D patients were younger, had lower Glasgow Coma Scale scores, fewer abnormal laboratory values, and were more likely to have neurological and traumatic disease. Reproducibility of the phenotypes was assessed by applying the same phenotype derivation process to the MIMIC-IV validation cohort; the clinical characteristics are presented in Table A3. The clinical characteristics of different phenotypes were also compared between the derivation cohort and the validation cohort, as shown in Figure A1. The results from the validation cohort confirmed that the optimal number of phenotypes, phenotype sizes, and clinical characteristics were similar to those observed in the derivation cohort.

### 3.3. Relationship between Phenotypes and Clinical Outcomes

**Figure 2:**
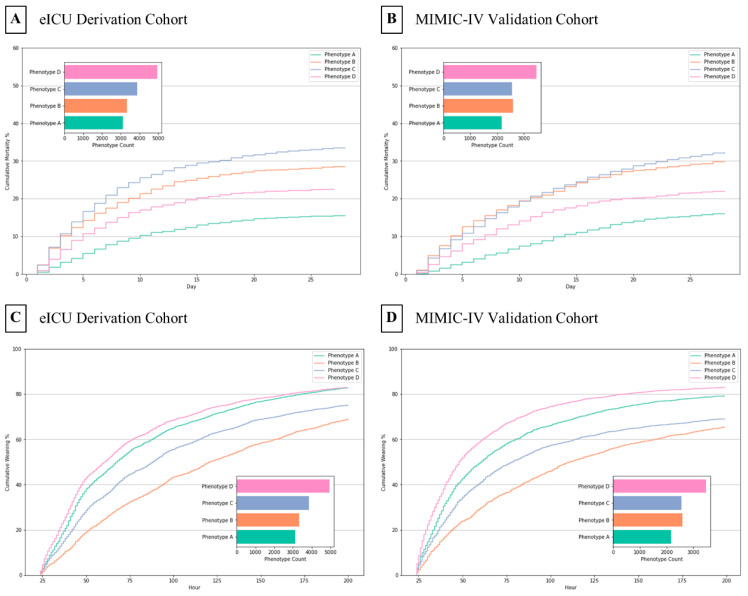
Cumulative mortality and extubation success rate by phenotypes. (**A**,**B**) 28-day mortality stratified by phenotype assignment. (**C**,**D**) Two hundred h extubation success rate stratified by phenotype assignment. The result suggests that clinical phenotypes can be generalized to another cohort.

The four derived phenotypes had different clinical outcomes, as shown in Figure 2. In both the derivation and validation cohorts, patients with phenotype A had the lowest 28-day mortality (approximately 15%) and a relatively high extubation success rate (approximately 80%); patients with phenotype B had the second-highest 28-day mortality (nearly 30%) and the lowest extubation success rate (approximately 65%); Phenotype C patients had the highest 28-day mortality (over 30%) and the second-lowest extubation success rate (about 70%); Phenotype D patients had the second-lowest 28-day mortality (just over 20%) and the highest extubation success rate (over 80%). Phenotype A had a significantly lower 28-day mortality compared to phenotype C, and phenotype D had a significantly higher extubation success rate than phenotype B. Patients assigned to phenotype B had the longest ICU length of stay (median: 7–9 days) and duration of mechanical ventilation (median: 3–4 days), whereas patients assigned to phenotype D had the shortest ICU length of stay (median: 5–6 days) and duration of mechanical ventilation (median: 1.5–2.5 days).

### 3.4. Heterogeneity of Treatment Effects

Among these four phenotypes, only phenotype B had a slightly higher proportion of patients requiring higher PEEP, and these patients had significantly lower extubation success rates (*p* < 0.05), longer ventilation duration (*p* < 0.05), and longer ICU stay (*p* < 0.05), but had no significant difference in hospital mortality (*p* = 0.248) (Table 2). Patients in phenotypes A, C, and D tended to require lower PEEP (*p* < 0.05), but had no difference in hospital mortality and extubation success rates. Patients with higher PEEP had longer ventilation duration in all four phenotypes and had longer ICU stays in phenotypes A, B, and C.

**Figure 3:**
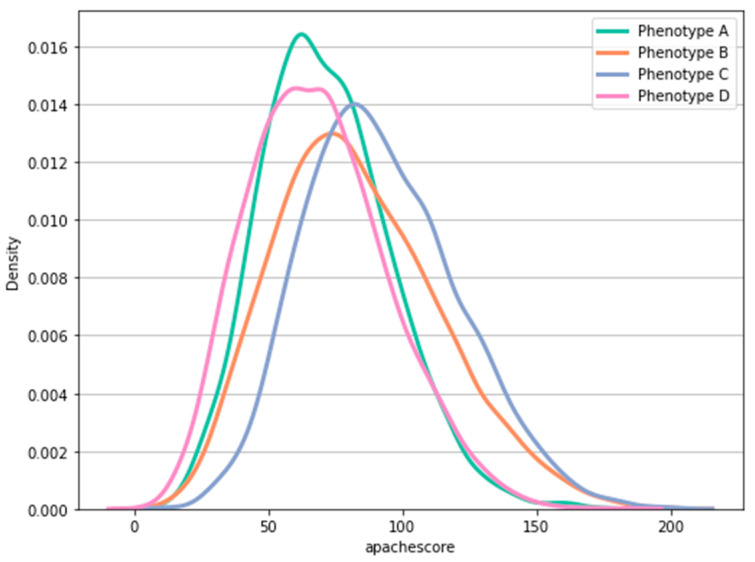
The estimated conditional distribution of APACHE IV score over phenotypes. Conditional distributions are estimated by using the eICU derivation cohort. Interpretation: conditional distributions across different phenotypes largely overlap, suggesting that clinical phenotypes do not simply reflect disease severity.

**Figure A2:**
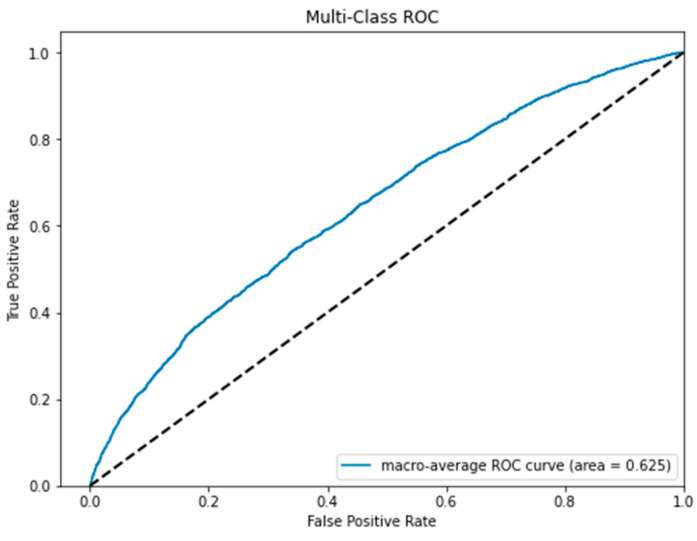
ROC AUC plot of the logistic regression model. ROC curve of phenotype classification model with APACHE IV score and RSBI as predictors.

Finally, we evaluated the relationship between the derived clinical phenotypes and the APACHE IV (Acute Physiology and Chronic Health Evaluation) score, as shown in Figure 3. The resulting plot confirmed that the conditional distribution of the different phenotypes largely overlapped, indicating that the clinical phenotypes are not a simple reflection of disease severity. In addition, we fitted a logistic regression model to classify the clinical phenotypes, using the APACHE score and RSBI as predictors. The low receiver operating characteristic (ROC) area under the curve (AUC = 0.625) of the model, as shown in Figure A2, indicated that the derived clinical phenotypes were not simply explained by the APACHE score or RSBI.

## 4. Discussion

This retrospective study used a large amount of data from two large publicly available databases, eICU and MIMIC-IV, which included large populations of heterogeneous mechanical ventilated ICU patients. Four clinical phenotypes were identified using only routinely available clinical data. The derived phenotypes were multidimensional, differed in demographics, vital signs, laboratory measures, and patterns of organ dysfunction, and could not be classified by simple stratification of diagnostic groups or severity of illness. In addition, these four phenotypes differed in mortality and other clinical outcomes. Although most of the clinical variables had different distributions in the derivation and validation cohorts (Table A2), the frequency and clinical characteristics of the phenotypes were reproducible in the MIMIC-IV validation cohort, demonstrating the universality of these four derived phenotypes. Only routinely available clinical variables were used in the clustering analysis, allowing the phenotypes to be easily reproduced in other datasets.

For these four phenotypes, we used chord diagrams to show correlations between phenotypes and clinical variable groups. Of the four phenotypes identified, phenotype A was correlated with abnormal values in pulmonary dysfunction; phenotype B was most strongly correlated with abnormal values associated with inflammatory, pulmonary, and cardiovascular dysfunction; phenotype C was most strongly correlated with renal dysfunction; and phenotype D had the least abnormal values. Cumulative mortality and extubation success rate plots showed that phenotype C had the highest mortality and phenotype B had the lowest extubation success rate. 

This study also showed that different clinical phenotypes responded differently to mechanical ventilation strategies. Most patients in phenotypes A, C, and D required lower PEEP, whereas phenotype B had a relatively higher proportion of patients that required higher PEEP. This may be due to pulmonary infiltration due to increased pulmonary capillary pressure during cardiac insufficiency. Additionally, patients in phenotype B who required higher PEEP had lower extubation success rates, longer ventilation times, and longer ICU stays. This suggests that patients with cardiovascular disease and respiratory dysfunction may require prolonged ventilation and intensive care. However, these four phenotypes were not statistically different in terms of mortality. In the era of personalized medicine, implementing different treatment strategies based on patient phenotypes could potentially improve treatment outcomes or help refine the ventilator weaning protocols.

These four phenotypes were identified on the first day of mechanical ventilation, aiming to help intensivists to identify different phenotypes of ICU patients early in the mechanical ventilation period, and potentially lead to different care processes for each phenotype. In eras and in regions where ventilators become a scarce resource, early prediction could not only help to avoid overtreatment but could also maximize the use of medical resources. Therefore, we only analyzed data on the first day of intubation, which may be controversial and one of our study’s limitations. To address this, our next study will develop predictive models using cross-sectional data on the day of extubation. Our second limitation was that the eICU database only included records from 2014 to 2015 and the MIMIC-IV database included records from 2008 to 2019. However, in the meantime, the weaning guideline changed in 2016, suggesting that the initial SBT should be performed with inspiratory pressure augmentation (5–8 cm H_2_O) rather than without (T-piece or CPAP). Therefore, we should validate the results using more recent cohorts which are not yet available. However, we reckoned that the revision of the guideline had little impact on this study, because the weaning process may vary according to patients, institutions, care processes, and the skills of different clinicians [21]. An international survey of intensivists’ weaning practices found that there were regional differences in several aspects such as screening frequency, SBT techniques, and ventilator mode [22]. The third limitation was that many decisions in the data preprocessing steps, such as the variable selection, time window selection, data cleaning, and data imputation methods, were made according to the other literature or our judgment. Therefore, there may be some bias in our results and changes in the decisions may lead to different clinical characteristics of the phenotypes.

## 5. Conclusions

In this retrospective study of mechanically ventilated ICU patients in the eICU and MIMIC-IV databases, four clinical phenotypes were identified which correlate with different clinical characteristics, and outcomes were identified. These phenotypes may help one to understand the heterogeneity of ICU patients, provide more personalized care and treatment strategies for patients, and ultimately lead to better survival and prognosis. Future research is needed to validate the phenotypes in different datasets and to determine the applicability of these phenotypes in clinical settings, including different weaning strategies and other care plans. 

## Figures and Tables

**Table 1:** Characteristics of the phenotypes in the derivation cohort.

Clinical Variables	Overall	Phenotype A	Phenotype B	Phenotype C	Phenotype D
n (%)	15,256	3112 (20.4%)	3335 (21.9%)	3868 (25.3%)	4941 (32.4%)
**Demographics**					
Age, median [Q1, Q3], years	64.0 [53.0, 74.0]	67.0 [58.0, 76.0]	60.0 [49.0, 70.0]	68.0 [58.0, 77.0]	61.0 [48.0, 72.0]
BMI, median [Q1, Q3]	28.0 [23.7, 33.9]	31.6 [25.8, 39.1]	28.9 [24.2, 35.4]	28.1 [24.0, 33.5]	25.9 [22.4, 30.4]
**Inflammation**					
Temperature, median [Q1, Q3], °C	36.9 [36.5, 37.4]	36.9 [36.6, 37.3]	37.1 [36.6, 37.6]	36.8 [36.3, 37.2]	36.9 [36.5, 37.4]
White blood cell count, median [Q1, Q3], ×10^9^/L	12.4 [8.9, 17.0]	11.3 [8.2, 15.0]	14.3 [10.2, 19.5]	13.4 [9.4, 18.6]	11.6 [8.6, 15.5]
**Pulmonary**					
Respiratory rate, median [Q1, Q3], breaths/min	19.0 [16.4, 22.1]	18.0 [16.1, 20.1]	22.9 [20.3, 26.1]	19.7 [17.0, 22.5]	17.0 [15.3, 19.4]
SpO_2_, median [Q1, Q3], %	98.0 [96.4, 99.2]	97.3 [95.9, 98.5]	96.3 [94.6, 97.7]	98.3 [96.9, 99.3]	99.1 [98.0, 99.7]
PaO_2_, median [Q1, Q3], mm Hg	127.0 [91.0, 184.4]	102.5 [79.7, 142.2]	99.0 [77.1, 133.0]	139.5 [99.3, 195.3]	164.0 [117.7, 222.0]
PaCO_2_, median [Q1, Q3], mm Hg	41.0 [35.2, 48.5]	55.3 [47.6, 65.7]	42.0 [36.5, 48.0]	37.5 [32.4, 43.0]	38.0 [34.0, 42.4]
Tidal volume, median [Q1, Q3]	502.9 [450.0, 557.7]	500.0 [450.0, 552.3]	500.0 [435.1, 550.0]	509.2 [456.8, 566.7]	505.6 [454.6, 560.6]
PEEP, median [Q1, Q3], cm H_2_O	5.0 [5.0, 7.1]	5.0 [5.0, 6.8]	8.2 [5.7, 10.0]	5.0 [5.0, 5.6]	5.0 [5.0, 5.0]
FiO_2_, median [Q1, Q3]	0.5 [0.4, 0.7]	0.5 [0.4, 0.6]	0.7 [0.6, 0.9]	0.5 [0.4, 0.6]	0.4 [0.4, 0.5]
**Cardiovascular**					
Heart rate, median [Q1, Q3], BPM	88.3 [76.6, 101.2]	82.4 [72.6, 93.2]	99.4 [86.7, 112.1]	89.5 [77.8, 101.6]	84.9 [73.7, 96.6]
Systolic blood pressure, median [Q1, Q3], mm Hg	113.6 [104.4, 125.6]	116.7 [107.4, 128.4]	108.2 [100.9, 117.6]	109.6 [101.6, 120.1]	119.5 [108.8, 132.5]
Bicarbonate, median [Q1, Q3], mmol/L	22.8 [19.9, 26.0]	28.5 [25.3, 33.0]	21.8 [19.1, 24.5]	19.6 [17.0, 22.2]	23.1 [21.0, 25.3]
**Hematologic**					
Red blood cell, median [Q1, Q3], ×10^9^/L	3.6 [3.1, 4.2]	3.9 [3.3, 4.4]	3.7 [3.2, 4.3]	3.2 [2.8, 3.7]	3.8 [3.3, 4.4]
Platelets, median [Q1, Q3], ×10^9^/L	191.5 [136.0, 254.5]	203.0 [152.0, 263.0]	195.0 [132.5, 263.0]	165.0 [109.5, 229.3]	199.5 [148.5, 261.0]
**Renal**					
Blood urea nitrogen, median [Q1, Q3], mg/dL	22.7 [14.3, 38.0]	24.0 [17.0, 36.0]	20.5 [14.0, 30.3]	45.0 [31.0, 63.5]	15.0 [10.7, 21.0]
Creatinine, median [Q1, Q3], mg/dL	1.1 [0.8, 1.9]	1.1 [0.8, 1.6]	1.1 [0.8, 1.6]	2.4 [1.6, 3.8]	0.8 [0.7, 1.1]
**Other**					
Glucose, median [Q1, Q3], mg/dL	140.8 [116.3, 174.0]	141.0 [118.0, 171.5]	145.0 [119.0, 181.0]	151.4 [123.0, 193.4]	131.3 [111.0, 156.7]
Potassium, median [Q1, Q3], mmol/L	4.0 [3.7, 4.5]	4.2 [3.9, 4.6]	4.0 [3.7, 4.4]	4.4 [4.0, 4.9]	3.8 [3.5, 4.0]
Sodium, median [Q1, Q3], mmol/L	139.4 [136.4, 142.5]	139.5 [137.0, 142.5]	139.0 [136.0, 142.5]	139.0 [135.4, 142.5]	140.0 [137.0, 142.5]
Calcium, median [Q1, Q3], mg/dL	8.2 [7.6, 8.7]	8.6 [8.2, 9.0]	7.9 [7.4, 8.4]	7.8 [7.2, 8.3]	8.3 [7.8, 8.7]
pH, median [Q1, Q3]	7.4 [7.3, 7.4]	7.3 [7.3, 7.4]	7.3 [7.3, 7.4]	7.3 [7.3, 7.4]	7.4 [7.3, 7.4]
Glasgow Coma Scale score, median [Q1, Q3]	8.8 [7.0, 10.3]	9.7 [8.0, 11.0]	9.0 [7.0, 10.5]	8.8 [6.9, 10.3]	8.1 [6.3, 9.8]

This table shows the characteristics of each derived clinical phenotype; all variables were significantly different between phenotypes (*p* < 0.001). Abbreviations: SpO_2_, oxygen saturation; PaO_2_, partial pressure of oxygen; PaCO_2_, partial pressure of carbon dioxide; FiO_2_, fraction of inspiration O2.

**Table 2:** Differences in response to PEEP strategy by phenotype (MIMIC-IV cohort).

PEEP Strategy	Phenotype A (n = 2175)		Phenotype B (n = 2604)		Phenotype C (n = 2567)		Phenotype D (n = 3467)	
	Low PEEP	High PEEP	Low PEEP	High PEEP	Low PEEP	High PEEP	Low PEEP	High PEEP
n (%)	1993 (91.6)	182 (8.4%)	1214 (46.6%)	1390 (53.4%)	2389 (93.1%)	178 (6.9%)	3428 (98.9%)	39 (1.1%)
Hospital Mortality	23.4%	23.6%	32.9%	35.2%	40.0%	46.1%	19.4%	23.1%
Extubation Success Rate	78.5%	72.5%	68.1% *	61.9% *	68.1%	62.9%	82.1%	76.9%
Ventilation Duration (Hours)	52.0 *	65.0 *	67.0 *	92.0 *	55.0 *	75.0 *	45.0 *	70.0 *
ICU Length of Stay (Hours)	178.8 *	204.7 *	193.2 *	234.6 *	169.0 *	213.7 *	147.7	147.1

* The values are statistically significant between low PEEP and high PEEP groups.

## Data Availability

Data are available upon request.

## Appendix A

**Table A1:** Characteristics of cohorts.

	Derivation Cohort	Validation Cohort
Database	eICU	MIMIC-IV
Number of patients	15,256	10,813
Extubation failure rate	23.1%	26.3%
28-day mortality	21.1%	24.1%

This table contains the basic characteristics of the two cohorts. Abbreviations: eICU, telehealth intensive care unit; MIMIC-IV, Medical Information Mart for Intensive Care.

**Table A2:** Comparison of clinical variables between two cohorts.

Clinical Variables	Missing	Overall	eICU	MIMIC-IV	*p*-Value
n (%)		26,069	15,256	10,813	
**Demographics**					
Age, median [Q1, Q3], years	0	64.0 [53.0, 74.0]	64.0 [53.0, 74.0]	64.0 [53.0, 75.0]	0.083
BMI, median [Q1, Q3]	1624	28.3 [24.0, 33.8]	28.0 [23.7, 33.9]	28.6 [24.6, 33.8]	<0.001
**Inflammation**					
Temperature, median [Q1, Q3], °C	758	37.0 [36.6, 37.4]	36.9 [36.5, 37.4]	37.0 [36.7, 37.4]	<0.001
White blood cell count, median [Q1, Q3], ×10^9^/L	424	12.3 [8.8, 16.9]	12.4 [8.9, 17.0]	12.2 [8.7, 16.7]	0.017
**Pulmonary**					
Respiratory rate, median [Q1, Q3], breaths/min	429	19.1 [16.6, 22.2]	19.0 [16.4, 22.1]	19.4 [17.0, 22.4]	<0.001
SpO_2_, median [Q1, Q3], %	2117	98.0 [96.4, 99.2]	98.0 [96.4, 99.2]	98.0 [96.4, 99.1]	0.006
PaO_2_, median [Q1, Q3], mm Hg	2640	123.0 [89.7, 173.8]	127.0 [91.0, 184.4]	120.0 [88.5, 164.8]	<0.001
PaCO_2_, median [Q1, Q3], mm Hg	2759	41.0 [36.0, 47.8]	41.0 [35.2, 48.5]	41.3 [36.9, 47.2]	<0.001
Tidal volume, median [Q1, Q3]	565	490.0 [429.9, 541.7]	502.9 [450.0, 557.7]	461.2 [409.9, 514.4]	<0.001
PEEP, median [Q1, Q3], cm H2O	1572	5.1 [5.0, 8.0]	5.0 [5.0, 7.1]	5.7 [5.0, 8.9]	<0.001
FiO_2_, median [Q1, Q3]	597	0.5 [0.4, 0.6]	0.5 [0.4, 0.7]	0.5 [0.4, 0.6]	<0.001
**Cardiovascular**					
Heart rate, median [Q1, Q3], BPM	75	87.3 [75.8, 99.9]	88.3 [76.6, 101.2]	85.8 [74.9, 98.2]	<0.001
Systolic blood pressure, median [Q1, Q3], mm Hg	726	112.5 [104.2, 124.0]	113.6 [104.4, 125.6]	111.5 [104.0, 121.8]	<0.001
Bicarbonate, median [Q1, Q3], mmol/L	88	22.7 [19.7, 25.9]	22.8 [19.9, 26.0]	22.5 [19.5, 25.5]	<0.001
**Hematologic**					
Red blood cell, median [Q1, Q3], ×10^9^/L	460	3.5 [3.0, 4.1]	3.6 [3.1, 4.2]	3.3 [2.9, 3.9]	<0.001
Platelets, median [Q1, Q3], ×10^9^/L	531	187.3 [130.0, 254.0]	191.5 [136.0, 254.5]	181.4 [121.8, 253.3]	<0.001
**Renal**					
Blood urea nitrogen, median [Q1, Q3], mg/dL	195	23.0 [14.8, 38.0]	22.7 [14.3, 38.0]	23.5 [15.2, 39.5]	<0.001
Creatinine, median [Q1, Q3], mg/dL	187	1.1 [0.8, 1.9]	1.1 [0.8, 1.9]	1.1 [0.8, 1.9]	0.174
**Other**					
Glucose, median [Q1, Q3], mg/dL	42	139.3 [115.0, 173.3]	140.8 [116.2, 174.0]	137.0 [113.2, 172.7]	<0.001
Potassium, median [Q1, Q3], mmol/L	124	4.1 [3.7, 4.5]	4.0 [3.7, 4.5]	4.2 [3.8, 4.6]	<0.001
Sodium, median [Q1, Q3], mmol/L	156	139.2 [136.3, 142.2]	139.4 [136.4, 142.5]	139.0 [136.2, 142.0]	<0.001
Calcium, median [Q1, Q3], mg/dL	854	8.2 [7.7, 8.7]	8.2 [7.6, 8.7]	8.2 [7.7, 8.7]	<0.001
pH, median [Q1, Q3]	2949	7.4 [7.3, 7.4]	7.4 [7.3, 7.4]	7.4 [7.3, 7.4]	<0.001
Glasgow Coma Scale score, median [Q1, Q3]	3434	12.3 [8.6, 15.0]	8.8 [7.0, 10.3]	15.0 [14.7, 15.0]	<0.001

This table contains the characteristics of each cohort; Kruskal–Wallis tests were performed; most variables were significantly different across the two cohorts (*p* < 0.001). Abbreviations: SpO_2_, oxygen saturation; PaO_2_, partial pressure of oxygen; PaCO_2_, partial pressure of carbon dioxide; FiO_2_, fraction of inspiration O_2_.

**Table A3:** Characteristics of the phenotypes in the validation cohort.

Clinical Variables	Overall	Phenotype A	Phenotype B	Phenotype C	Phenotype D
n (%)	10,813	2175 (20.1%)	2604 (24.1%)	2567 (23.7%)	3467 (32.1%)
**Demographics**					
Age, median [Q1, Q3], years	64.0 [53.0, 75.0]	69.0 [59.0, 79.0]	60.0 [49.8, 70.0]	67.0 [57.0, 77.0]	60.0 [47.0, 72.0]
BMI, median [Q1, Q3]	28.6 [24.6, 33.7]	27.2 [23.4, 32.5]	32.9 [28.2, 39.3]	28.7 [25.0, 33.3]	26.7 [23.3, 30.7]
**Inflammation**					
Temperature, median [Q1, Q3], °C	37.0 [36.7, 37.4]	37.0 [36.7, 37.4]	37.2 [36.8, 37.6]	36.8 [36.4, 37.1]	37.1 [36.8, 37.5]
White blood cell count, median [Q1, Q3], ×10^9^/L	12.2 [8.8, 16.8]	11.3 [8.2, 15.1]	14.6 [10.3, 20.0]	12.6 [8.9, 17.8]	11.2 [8.3, 14.8]
**Pulmonary**					
Respiratory rate, median [Q1, Q3], breaths/min	19.5 [17.0, 22.4]	19.2 [17.2, 21.3]	23.3 [20.6, 26.2]	19.6 [17.2, 22.1]	17.4 [15.7, 19.5]
SpO_2_, median [Q1, Q3], %	98.0 [96.4, 99.1]	97.5 [96.3, 98.6]	96.0 [94.4, 97.4]	98.3 [97.0, 99.2]	99.0 [98.0, 99.6]
PaO_2_, median [Q1, Q3], mm Hg	120.2 [88.7, 165.6]	89.7 [65.9, 115.7]	99.8 [81.3, 126.5]	129.0 [97.0, 167.0]	163.7 [123.3, 211.8]
PaCO_2_, median [Q1, Q3], mm Hg	41.5 [37.0, 47.2]	50.0 [44.3, 58.5]	43.4 [39.0, 49.6]	38.8 [35.0, 43.0]	38.9 [35.2, 42.5]
Tidal volume, median [Q1, Q3]	460.6 [410.4, 513.9]	422.6 [372.1, 468.4]	465.9 [416.5, 519.7]	472.0 [419.2, 522.6]	474.5 [427.4, 525.2]
PEEP, median [Q1, Q3], cm H2O	5.6 [5.0, 8.9]	5.6 [5.0, 8.0]	10.2 [8.5, 12.5]	5.4 [5.0, 7.7]	5.0 [5.0, 5.5]
FiO_2_, median [Q1, Q3]	0.5 [0.4, 0.6]	0.5 [0.4, 0.6]	0.7 [0.6, 0.7]	0.5 [0.4, 0.6]	0.5 [0.4, 0.5]
**Cardiovascular**					
Heart rate, median [Q1, Q3], BPM	85.9 [75.1, 98.2]	81.5 [72.4, 92.9]	96.1 [84.5, 107.7]	84.9 [74.5, 96.6]	82.2 [72.7, 93.9]
Systolic blood pressure, median [Q1, Q3], mm Hg	111.7 [104.1, 121.9]	113.5 [105.9, 123.4]	106.6 [100.2, 113.7]	109.6 [103.3, 118.2]	117.3 [108.5, 129.5]
Bicarbonate, median [Q1, Q3], mmol/L	22.3 [19.5, 25.5]	27.0 [24.3, 30.7]	21.0 [18.3, 24.0]	19.3 [16.8, 22.0]	23.0 [21.0, 25.0]
**Hematologic**					
Red blood cell, median [Q1, Q3], ×10^9^/L	3.3 [2.9, 3.9]	3.3 [2.9, 3.8]	3.6 [3.1, 4.1]	3.0 [2.7, 3.4]	3.5 [3.1, 4.0]
Platelets, median [Q1, Q3], ×10^9^/L	182.0 [123.0, 253.3]	209.0 [151.1, 289.0]	186.7 [126.0, 256.8]	143.5 [91.8, 209.3]	189.0 [135.0, 255.5]
**Renal**					
Blood urea nitrogen, median [Q1, Q3], mg/dL	23.5 [15.2, 40.0]	23.5 [16.5, 35.0]	24.5 [16.8, 38.0]	48.0 [32.0, 68.0]	15.2 [11.0, 21.0]
Creatinine, median [Q1, Q3], mg/dL	1.1 [0.8, 1.9]	1.0 [0.7, 1.4]	1.3 [0.9, 2.0]	2.5 [1.6, 3.9]	0.8 [0.6, 1.1]
**Other**					
Glucose, median [Q1, Q3], mg/dL	137.0 [113.0, 173.0]	131.0 [109.0, 161.4]	151.0 [123.4, 196.1]	146.8 [115.5, 186.8]	128.5 [109.0, 155.0]
Potassium, median [Q1, Q3], mmol/L	4.2 [3.8, 4.6]	4.1 [3.8, 4.5]	4.3 [3.9, 4.7]	4.5 [4.1, 5.0]	3.9 [3.7, 4.2]
Sodium, median [Q1, Q3], mmol/L	139.0 [136.2, 142.0]	140.3 [137.5, 143.3]	138.5 [135.5, 141.5]	138.0 [134.4, 140.8]	139.7 [137.3, 142.5]
Calcium, median [Q1, Q3], mg/dL	8.2 [7.7, 8.7]	8.4 [8.0, 8.9]	7.9 [7.5, 8.4]	8.2 [7.7, 8.7]	8.2 [7.8, 8.7]
pH, median [Q1, Q3]	7.4 [7.3, 7.4]	7.4 [7.3, 7.4]	7.3 [7.3, 7.4]	7.3 [7.3, 7.4]	7.4 [7.4, 7.4]
Glasgow Coma Scale score, median [Q1, Q3]	15.0 [14.7, 15.0]	15.0 [14.7, 15.0]	15.0 [14.6, 15.0]	15.0 [14.6, 15.0]	15.0 [14.7, 15.0]

This table contains the characteristics of each derived clinical phenotype in the validation cohort; all variables were significantly different across phenotypes (*p* < 0.001). Abbreviations: SpO_2_, oxygen saturation; PaO_2_, partial pressure of oxygen; PaCO_2_, partial pressure of carbon dioxide; FiO_2_, fraction of inspiration O_2_.
